# Emigration dynamics of cockroaches under different disturbance regimes do not depend on individual personalities

**DOI:** 10.1038/srep44528

**Published:** 2017-03-16

**Authors:** I. Planas-Sitjà, M. O. Laurent Salazar, G. Sempo, J. L. Deneubourg

**Affiliations:** 1 Unit of Social Ecology - CP 231, Université libre de Bruxelles (ULB), Campus Plaine, Boulevard du Triomphe, Building NO - level 5, B-1050 Bruxelles, Belgium

## Abstract

Group-level properties, such as collective movements or decisions, can be considered an outcome of the interplay between individual behavior and social interactions. However, the respective influences of individual preferences and social interactions are not evident. In this research, we study the implications of behavioral variability on the migration dynamics of a group of gregarious insects (*Periplaneta americana*) subjected to two different disturbance regimes (one without disturbances and another one with high frequency of disturbances). The results indicate that individuals presented consistent behavior during the nighttime (active phase of cockroaches) in both conditions. Moreover, we used a modeling approach to test the role of personality during the migration process. The model considers identical individuals (no personality) without memory and no direct inter-attraction between individuals. The agreement between theoretical and experimental results shows that behavioral variability play a secondary role during migration dynamics. Our results showing individual personality during the nighttime (spontaneous decision to forage) but not during the emigration process (induced by environmental disturbances) highlight the plasticity of personality traits.

A large community of researchers has been inspired by the coordinated movements and decision-making, including insect colonies, fish schools, bird flocks and mammal herds^1^,^2^,^3^,^4^,^5^. One important aspect of social species is that the animals routinely face crucial decisions that must be made simultaneously with other group members to reach the most accurate choice^6^, and the methods by which individual behavior and social interactions produce group-level behavior is at the heart of collective behavior research^7^,^8^,^9^,^10^. Indeed, the outcome of group behavior emerges from individual decisions in response to environmental stimuli and to cues or signals from other group members^11^. For instance, when choosing habitats in a patchy environment, group-living species are confronted with a choice between many sites that may differ in their intrinsic quality^12^. The success of collective decisions depend on the accuracy of the information and the preferences of the individuals. In such cases, social information (such as the presence of conspecifics) can provide a local social cue^13^ that can be used by individuals to accurately estimate the habitat quality^14^,^15^,^16^,^17^. Moreover, the presence of conspecifics can improve the fitness of individual group members through cooperation^18^.

Recently, researchers have focused on another aspect of collective movements and decisions: the reactions of groups when subjected to disturbances. With increases in human populations, one of the primary interests of researchers is the impact of anthropogenic disturbances on animal populations, including group-living species^19^,^20^,^21^,^22^. Well-known studies have explained how these disturbances affect animal behavior and reproductive success and therefore impact the ecology and evolution of these species^23^,^24^,^25^,^26^. The responses to disturbances vary depending on the animals’ overall ecological landscape^24^,^27^. Moreover, the foraging patch quality, social context and individual’s condition and previous history can influence the onset and intensity of any response^28^. As a result of such sources of information, individuals within a group can vary in their responses to a disturbance and may tolerate the disturbance or flee from the disturbed areas^29^. For instance, studies of termites have reported that their escape from a disturbed location was usually temporary and indicated that the time required to return to the disturbed area varied depending on the termite species, disturbance type, soldier proportions and environmental factors, such as temperature^30^,^31^,^32^. Despite the obvious impacts of disturbances to wildlife, relatively few studies have focused on the collective dynamics of responses to environments with frequent disturbances.

The major problem of studying group dynamics is the difficulty of dissociating social effects from individual threshold responses and habituation^33^ because from an observational perspective, all of these factors can result in same outcome. For instance, if a group’s response to repeated disturbances decreases over time, it may be due to (A) a habituation process, (B) a group size effect (i.e., certain individuals emigrate and the size of the group diminishes along with social interactions; therefore, individuals can decrease the reaction to the disturbance) or (C) group divisions along personality lines (i.e., bold individuals emigrate while shy individuals remaining; thus, the individuals who remain will show diminishing reactions to the disturbance). Moreover, these hypotheses, among others, might act concomitantly in nature to explain how a group responds to a disturbance event. Understanding how these factors, which may be key drivers of group success, impact collective behavior will improve our understanding of animal societies.

Group size and personality have been reported to play important roles in foraging, decision making, escape behavior and vigilance in several species^34^,^35^,^36^,^37^,^38^. In this study we analyze the effects of disturbances on group behavior and how it depends on the synergy between sociality and personality. For this purpose, we use an insect model for group-living studies, the American cockroach (*Periplaneta americana*), which has been shown to emigrate from disturbed shelters to non-disturbed shelters^33^. Social behavior and group size effects have already been described in cockroaches presenting collective decision making^17^,^39^,^40^,^41^, and individuals have shown consistent differences during the aggregation process^42^. However, Laurent Salazar *et al*.^33^ showed that social interactions played a negligible role for the exploring behavior of cockroaches during the active phase (nighttime). This exploring behavior is a mainly solitary activity in cockroaches. Our hypothesis, is that the emigration process in an environment with frequent disturbances is strongly affected by individual personalities and amplified by social interactions (i.e., the individual probability of fleeing will increase along with the number of individuals leaving).

Our hypothesis is that the synergy between the consistency of individual behavior (stable inter-individual differences) and social interactions plays a key role during the migration induced by frequent disturbances. We therefore propose a stochastic model to predict the spatial dynamics in our control condition (undisturbed environment) and an environment with frequent disturbances. If our hypothesis is true, the model results will likely differ because of the lack of sensitivity in the individual behavioral differences. Indeed, this model, which neglects stable inter-individual differences (i.e., considers individuals to be identical units with mean probabilities determined from the experimental results), should be unable to reproduce the migration pattern observed in an environment with frequent disturbances. However, we expect that this model will be able to reproduce the stability of shelter choice in the control condition, as behavioral consistency should play a minor role in this condition. If the model can provide an accurate estimate of the spatial dynamics in an environment with frequent disturbances, then the role of individual behavioral stability during the emigration process must be reconsidered.

## Results

### Personality and group size effects during the active and resting phases

Groups of 20 cockroaches were tested in an arena with two identical shelters for a week (see methods). In the control or undisturbed condition (UD), the shelters remained undisturbed, while in the condition with frequent disturbances (D) the selected shelter for cockroaches on the first day was daily disturbed with light. First, we analyzed the behavioral consistency of cockroaches during the active phase (nighttime) when they explored the arena. For this purpose we took into account different parameters that have been previously described to be good proxies of shelter use and foraging activity. Cockroaches showed significant behavioral consistency for all of the parameters explored (Table 1) in the UD and D conditions. Indeed, the Kendall coefficients obtained for the Total Time Outside (TTO) and other temporal measures, such as the duration of the first exit, were greater than that expected by the Kendall random distribution (KRD), which assumes that all individuals are identical. High values of Kendall coefficient, ranging from 0 to 1, indicate consistency between days (see methods).

**Figure 1:**
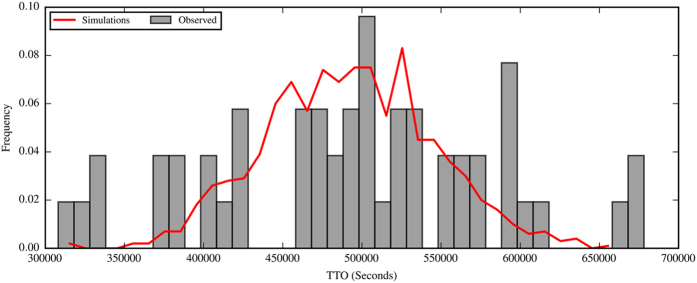
Comparison between the groups’ TTO of the experimental groups (grey bars) and the groups’ TTO of the artificial groups (red line).

Furthermore, we analyzed whether different social dynamics occurred within the groups. For this purpose, we created artificial or simulated groups from the experimental data. Each simulated group was composed of 20 individuals randomly selected from the pool of tested individuals’ data (120 for the D condition and 140 for the UD condition). Using this method, individuals from the artificial groups maintained their behavioral consistency (its own TTO for 4 nights) but were mixed among groups. We compared the sum of the individuals’ TTO within experimental groups with the sum of TTO of the artificial groups (Fig. 1), and significant differences were not observed between the distributions (KS Test: D_52,200_ = 0.26462, P = 0.3014), which indicated the absence of different social dynamics within groups because mixing the individuals’ TTO did not affect the groups’ TTO distribution. If the groups had presented different social dynamics, we should have observed changes in the groups’ TTO distribution after mixing individuals from the different groups.

Regarding the resting phase (daytime), we analyzed the effect of group size during the disturbances, and correlations were not observed between the time required to leave the S shelter after a disturbance and the size of the group at the beginning of the disturbance (regression test for the first disturbance of each day: R^2^ = 0.01, F_1,233_ = 73.63, P = 0.58), which indicated that the effect of group size on emigration behavior is negligible. A habituation process was not observed, and after a greater number of perturbations, individuals did not decrease their reaction time. Indeed, the mean individual time required to leave the shelter during disturbances was not correlated with the number of disturbances impacting the same individual (Kendall correlation test: R = 0.06, τ = 0.89, N = 113, P = 0.37).

### Modeling emigration dynamics and choice stability in the disturbed and undisturbed conditions

**Figure 2:**
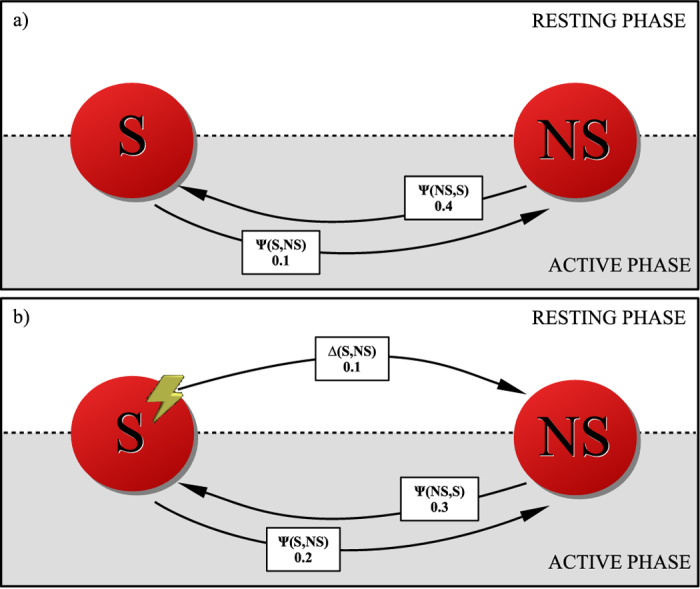
Schema representing the probabilities of visiting a certain shelter during the active and inactive phases: (**a**) Undisturbed condition; and (**b**) Disturbed condition.

We propose a stochastic model in aim to predict the cockroaches’ behavior over time and test whether a group of identical individuals without individual memory reproduce the global emigration dynamics and the behavioral response variance. Therefore, the model needs the mean probability of leaving and joining a shelter during the active and resting phases. For this purpose, we used the experimental results to quantify the fraction of individuals from all groups (UD and D condition separately) that were in a shelter at the beginning of a given phase and moved to another shelter at the end of it (active or resting phase). We calculated these mean probabilities for the Selected (S) and Non-Selected (NS) shelters during both phases and we implemented them in our model. The initial state for this model was asymmetric, which was the same as our observations because most cockroaches were under the S shelter at the beginning of the experiments. The same model was performed for the UD and D conditions with the respective probability values (see Fig. 2). In addition, for the D condition we show that individual’s TTO did not influence the transition occurrences between shelters. In other words, that the individuals transitioning from one shelter to the other or to the same one during the active phase, showed no significantly different TTO (see Table 2). Regarding the UD condition, due to the high number of occurrences between S to S shelter against other possible transitions (S to NS, NS to NS and NS to S), we could not perform any reliable statistical test.

Figure 2 shows a diagram based on our results that describes the spatial dynamics of cockroaches over 24 h (resting + active phase) for the UD and D conditions. As the TTO did not influence the transition between shelters, this model neglects the nocturnal activities (such as TTO) and is only based on the individual probabilities of transition between shelters. Regarding the UD condition (Fig. 2a), the cockroaches never spontaneously left the shelters during the resting phase; therefore, the only movement between shelters occurred during the active phase. During this phase, there was a probability of moving to the opposite shelter, with Ψ(S, NS) and Ψ(NS, S) representing the probabilities of moving from the S to the NS shelter and from the NS to the S shelter during the night, respectively. In addition, Ψ(NS, NS) (=1−Ψ(NS, S)) and Ψ(S, S) (=1−Ψ(S, NS)) are the probabilities of returning to the previously occupied shelter. In our experimental case, the individual probability Ψ(NS, S) was greater than its opposite, meaning that the S shelter was more attractive than the NS shelter (Ψ(S, NS) = 0.1; Ψ(NS, S) = 0.4, KS test: D_1,3_ = 1, P = 0.01; Fig. 2a).

Concerning the D condition (Fig. 2b), the schema becomes more complex because of the cockroaches moving between shelters during the resting phase. During the active phase, the cockroaches were able to move between the S and NS shelters, which was similar to the UD condition, although these probabilities for the D condition were not significantly different (Ψ(NS, S) = 0.3 and Ψ(S, NS) = 0.2, KS test: D_1,3_ = 0.5, P = 0.53). A comparison of both conditions indicated that the Ψ(NS, S) was not significantly different (KS test: D_1,3_ = 0.5, P = 0.53). Nevertheless, the Ψ(S, NS) was greater for the D condition than the UD condition (KS test: D_1,3_ = 1, P = 0.01, see Fig. 2). Regarding the resting phase, individuals in the NS shelter did not move spontaneously, like in the UD condition. However, cockroaches in the S shelter had a probability of moving to the NS shelter when subjected to a disturbance. The probability of having moved (Δ(S, NS)) and remaining in the S shelter (Δ(*S, S*)) by the end of the resting phase are determined by eqs (1) and (2) respectively. In the UD condition, Δ(*S, NS*) = 0; and in the D condition, Δ(*S, NS*) = 0.1. In this case (eqs 1–2), *θ* is the conditional probability of moving between the S and NS shelter for each disturbance and *l* is the number of disturbances per day.


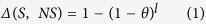



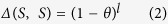


The experimental evolution of the number of cockroaches that settled inside each shelter in the UD and D conditions is reproduced by a simulation and eqs (3–5) (comparison with the observed results: KS test: D_1,4_ = 0.4, P = 0.7). Eqs (3–5) considers the different transition probabilities between the S and NS shelters. *P(n*) (*Q(n*)) is the probability of cockroaches settling for the resting phase in the S (NS) shelter at day *n* and depends on the probabilities of moving during the resting and active phase. *Tr* is the resulting probability of remaining in the S shelter, as day *n*-1, and is related to the disturbance responses and the nocturnal activity of the cockroaches.


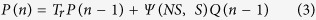



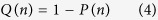



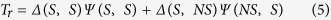


The solution of eqs (3–5) is as follows:


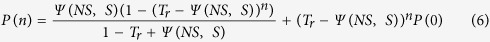


The stationary state is given by eq. (7):


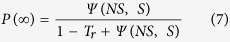


**Figure 3:**
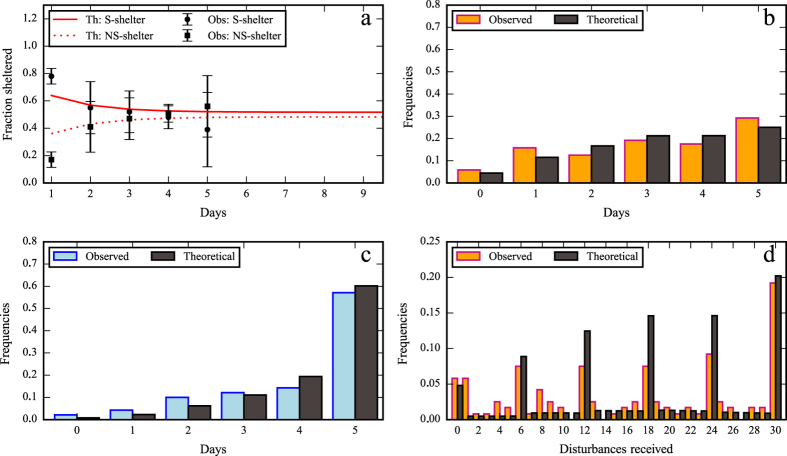
Comparison between our observations and the theoretical results. (**a**) Observed and theoretical mean fraction of individuals sheltered inside each shelter throughout the study period during the day (±SD). Frequency of the number of days each individual was found under the Selected (S) shelter in (**b**) the Disturbed and (**c**) Undisturbed condition. (**d**) Frequency of the total number of disturbances experienced by an individual.

In the D condition, diurnal disturbances cause the population in the S shelter to diminish each day and the population in the NS shelter to increase until it reaches a value at the end of the week that is close to stationary state (=0.54) predicted by the model after 5 days (see eq. 7 and Fig. 3a). In addition, eq. (7) predicts that greater Δ(S, NS) values lower the probability of residing in the S shelter at the stationary state, which means that the final proportion of the sheltered cockroaches in the S shelter will be close to Ψ(NS, S). In the UD condition, which does not present a diurnal migration (Δ(S, NS) = 0), *Tr* is equal to Ψ(S, S). Interestingly, the mean proportion of cockroaches that settled in the S shelter at the beginning of the experiment was already close to the predicted stationary state predicted by the model in the UD condition (=0.8). This agreement suggests that without disturbances the stationary state is rapidly reached from the first day.

Furthermore, we used eqs (8–9) to estimate the number of individuals in the S shelter at the beginning of each resting phase. The probability of settling in the S shelter or NS shelter (*P(n, i*) and *Q(n, i*), respectively) at the beginning of day *n* and with *i* previous days in the S shelter is given by eqs (8–9).


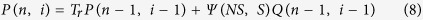



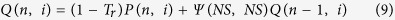


The theoretical distribution of the total probability (*P(n, i*) + *Q(n, i*)) is similar to the experimental results for the UD and D conditions (KS test: D_1,5_ = 0.17, P = 1 and KS test: D_1,5_ = 0.33, P = 0.93, respectively, Fig. 3b,c). For both conditions, the number of cockroaches that spend between 5 and 0 days under the selected shelter decreases in a continuous way (Fig. 3b,c). This lack of differences between our model that considers identical individuals and our experimental frequencies strongly suggest that personality did not have an effect on site fidelity.

For the D condition, we analyzed the total number of disturbances experienced by each individual (Fig. 3d). Indeed, most of the cockroaches resting in the S shelter experienced all 6 disturbances per day. The probability of settling in the S shelter (*P*^*E*^ (*n, k*)) or NS shelter (*Q*^*E*^ (*n, k*)) at the end of day *n* after experiencing *k* disturbances is as follows (see SI 1 for a detailed explanation):


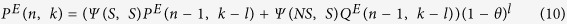



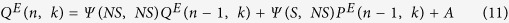



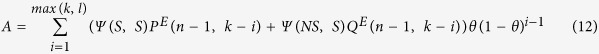


The theoretical results obtained with eqs (10–12) and the observed disturbances experienced by each individual (Fig. 3d) are correlated. Moreover, the theoretical distribution of the number of disturbances experienced at the end of each resting phase does not vary from the experimental distribution (KS test: D_1,5_ = 0.5, P = 0.44; see SI 2).

## Discussion

### Nighttime dynamics and the role of personality

Our results show that stable inter-individual differences can be observed during nighttime but they are not observed during the migration process induced by frequent disturbances during the daytime. Regarding the active phase (nighttime), we measured the Total Time Outside (TTO) and a series of punctual events, such as the duration and the timing of the first exit, which showed a high inter-individual behavioral consistency over the week (Table 1).

These findings complement the results obtained in previous studies on domiciliary cockroach personalities and their effects on collective behavior^33^,^42^,^43^. Indeed, Laurent Salazar *et al*.^33^ showed that behavior during the active phase could be explained without the use of social interactions. These results are consistent with the results of our study, in which the behavioral consistency observed during trials did not arise from group-level effects because a similar consistency was observed in artificially reshuffled groups as in experimental groups (see Fig. 1). Thus, we conclude that group personality (consistent between-groups behavioral differences) is not caused by different social interactions within groups and can only be achieved if individuals display consistent behavior. This finding supports studies that have focused on key individuals and the methods by which an individual’s personality can drive the colony/group personality, which has been observed in other arthropods^44^.

Our results show that individuals behave consistently during the active phase, although how this behavioral consistency affects the emigration process remains unclear. Our hypothesis suggested that personality will have an effect on emigration dynamics, which has been demonstrated in spiders, for which personality was a better determinant of the collective foraging success than others factors^37^, and habitat alteration may have an effect on the group personality^45^. Therefore, an evolutionary perspective indicates that daily disturbances could divide the population^46^ by selecting for shy personalities to remain sheltered and bold individuals to emigrate to a better shelter. Indeed, the foraging patch quality and social context as well as the individual’s condition and its previous encounters with specific stressors can all influence the onset and intensity of any response^28^,^47^. As a result of such trade-offs, individuals might decide to tolerate a disturbance rather than flee from disturbed areas^29^. Over a long time scale, this phenomenon could generate two different populations that would show different collective dynamics^42^. We built a probability model to test the role of personality in the stability of aggregates (UD condition) and dynamics of emigration (D condition).

### Modeling emigration dynamics and choice stability in the disturbed and undisturbed conditions

The model that only considers equal individuals (without personality) predicts the stability observed in a group of cockroaches, the disturbance-induced emigration dynamics, and the variance of the behavioral responses, and these results invalidate our previous hypothesis that personality was the key driver of the emigration process. In addition, domiciliary cockroaches are subjected to social interactions during the aggregation and consensus decision-making processes^17^,^40^,^41^,^48^. Nevertheless, a recent article showed that these interactions are not necessary to explain the active nocturnal behavior of cockroaches^33^. In this study, the groups had already made a collective decision before the first day of experiment. Hence, the model did not consider direct inter-attraction between individuals and only considered the exploratory behavior during the active phase and the settlement process during the resting phase, which is affected by hydrocarbon deposition (indirect inter-attraction)^49^. Indeed, the probability Ψ(NS, S) was greater than the Ψ(S, NS) for the UD condition, thus favoring the occupation of the S shelter. This result agrees with past studies showing that greater hydrocarbon concentrations inside a shelter increased the attractiveness of the shelter^49^.

Regarding the D condition, the probabilities of moving between shelters (Ψ(NS, S) and Ψ(S, NS)) had similar values, contrarily to the UD condition. These differences between both conditions suggest that hydrocarbons marking the substrate play an important role in the stability of the aggregate. We assume that individuals had the same probabilities at the beginning of the experiments in the UD and D conditions because the cockroaches had not experienced prior disturbances. Following the disturbances, individuals emigrated from the S to the NS shelter, and the increased group size in the NS shelter increased the attractiveness (i.e., the concentration of hydrocarbons) of this shelter. Without such evolution of the probabilities of moving between shelters, a complete emigration would not succeed.

From an ecological perspective, cuticular hydrocarbons play an important role during the collective emigration towards a new shelter in domiciliary cockroaches. Nevertheless, this emigration process towards a new shelter occurs slowly. Hence, for an indeterminate interval, the aggregate diminishes in size, which increases the risks associated with such a decrease^50^. In addition, because the probability of staying in the same shelter during the active phase is greater than the probability of moving to the other shelter, we conclude that there is an individual memory or individual fidelity with regard to the shelters. However, these probabilities cannot be considered different between individuals. In other words, hydrocarbon marking acts as a global memory that drives population stability.

The model is able to reproduce disturbance-induced behaviors and gradual emigration dynamics. It neglects direct social interactions and only considers the probability of leaving during a disturbance and the probability of moving from one shelter to the other during the active phase (see Figs 2 and 3). Therefore, because the model only considers equal individuals, we conclude that personality does not affect the emigration process. Nevertheless, these results do not indicate that the behavioral consistency disappears during the resting phase; rather, it is overshadowed by the global emigration dynamics that occur during disturbances. Indeed, male cockroaches in a sleep-like state can be individually recognized by their specific respiratory behavior (P. Kestler, pers. com.). In such cases, behavioral consistency can be considered context dependent. The absence of personality during the disturbances can be explained by a different threshold response that occurs among individuals but it is overshadowed by the magnitude of the disturbance. A further experiment with different disturbance intensities could provide insights on this hypothesis. In several species, reaction times are influenced by the group size^3^,^41^,^51^,^52^, although in the current study, we observed that the time required to leave a shelter after a disturbance was not affected by the group size. These results are not inconsistent because reaction times depend on the group size but not the time required to exit the shelter^41^.

In this study, we show how groups that had selected a shelter the night before the experiment, were able to sense the degradation of the shelter quality and migrate to another one. The individual fitness of cockroaches has been reported to increase with the group size^53^,^54^. Therefore, the fitness of the emigrating cockroaches was dependent on the quality of the shelter (disturbed or not) and the size of the group. Individuals must be sensitive to both factors and react according to the trade-offs between staying in the disturbed shelter or moving towards the non-disturbed shelter, which brings increased risk. From an ecological and evolutionary perspective, the most fit communities are those capable of emigrating towards the NS shelter and maintaining the group size, which indicates that most of the group will have to emigrate together. However, during a slow emigration, cockroaches will be distributed between shelters, thereby reducing the group size. A diminishing group size might not have an effect in an environment exempt of predation risk, such as in birds, which show a tendency to reduce the flocking size on islands with relaxed predation^55^. Nevertheless, the individual fitness of species that benefit from aggregation (e.g., decreased physical stress, increased foraging and reproductive success or improved collective decisions) will be affected by decreases in the group size^34^.

In the present case, the composition of personalities within a group has a minor effect during the emigration process, which means that the important factor is the ability to emigrate at the group level regardless of the individuals within the group. Finally, we hypothesize that the observed personalities in males of the same age are related to different activity patterns (i.e., variability in activity patterns, such as locomotion or respiration) or threshold response levels (i.e., response to light or electric stimuli). New experiments that integrate genetic variability and epigenetics as well as physiological analyses with behavioral assays could provide insights into the behavioral plasticity and personality effects of social insects.

## Methods

### Biological model

*Periplaneta americana* follow a circadian rhythm that is characterized by an active phase (nighttime) and resting phase (daytime), during which it forms aggregates in dark, warm, and damp locations^56^. Individuals were reared in the Université libre de Bruxelles in Plexiglas vivaria and provided with dog pellets and water *ad libitum*. The rearing room was maintained at 25 °C ± 1 under a 12 h:12 h L:D cycle.

### Setup

**Figure 4:**
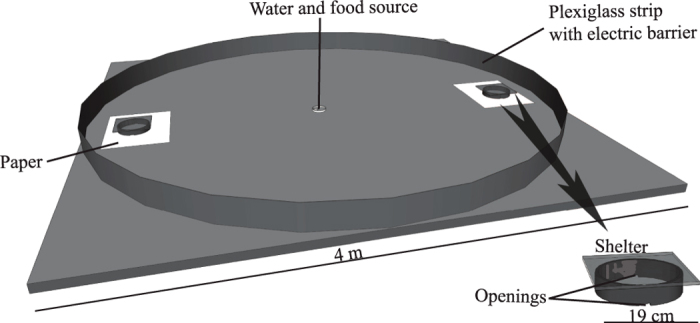
Experimental setup. The shelters were placed 40 cm from the Plexiglas strip. The two shelter openings were opposite each other and facing away from the water and food source.

The experiments were conducted in a circular arena (4 m Ø) limited by a 20 cm high Plexiglas strip and an electric fence (19 V, 0.2 A) (Fig. 4; see ref. 33 for more details). The shelters were constructed of two plastic rings (19 cm in diameter and 4.5 cm in height) that were covered by one sheet of a red-colored filter (Rosco ^®^ E-Colour #19:fire) and then placed 40 cm from the Plexiglas on opposite sides of the arena. The shelters had two openings (3 × 1.5 cm) placed symmetrically on opposite sides and facing away from the food and water source, which were placed at the center of the arena. White paper (120 g/m^2^) was placed under the shelters and changed after each experiment to prevent any chemical marking between experiments^49^. The interior face of the glass covers was lined with diffused white LEDs. When these LEDs were turned on, only the interior of the shelters were illuminated (λd: 468.3 nm; Ev: 308.41 lx), thereby forcing the individuals to flee the shelter.

The experiments were conducted in a room maintained at 25 °C ± 1 under a 12 h:12 h LD cycle (08:00–20:00; 20:00–08:00). To detect the cockroaches inside a shelter, a circular RFID lecture panel was situated under the shelters, and each individual was tagged on the pronotum with a RFID chip (diameter, 7.1 ± 0.2 mm; weight, 107 ± 3 mg; Spacecode^®^), which was glued in place by a drop of latex (Winsor & Newton^®^).

### Experimental procedure and measurements

From the rearing room, we isolated into plastic containers (36 × 14 × 24 cm) adult male cohorts of the same age that were without external damage and had undergone their imaginal molting during the same month. The cockroaches had access to a piece of water-soaked cotton and dog pellets (Tom & Co.^®^). On the evening preceding the start of the experiment, we tagged the individual cockroaches with an RFID chip.

Twenty individuals were introduced into the arena one hour after tagging at approximately 19:00 h. The experiments started at 08:00 h the next day and lasted for 5 days and 4 nights. During each experiment, the RFIDs recorded (approximately every 3 seconds) (a) the number of individuals inside each shelter at every moment, (b) the total amount of time spent inside/outside shelters and (c) the number of visits to each shelter.

In the Disturbed condition (N = 6), we disturbed the shelter in which the majority of the individuals were aggregated at 08:00 h the first day (more than half of the individuals in the aggregation). Hereafter, this shelter will be referred to as the selected shelter (S) and the other shelter will be referred to as the non-selected shelter (NS). If the shelters are denoted as “left” and “right”, if the majority of individuals had aggregated inside the “left” shelter, then this shelter would be the only shelter that was disturbed, regardless on the number of cockroaches aggregated there through the remainder of the trial. The shelter was disturbed 6 times per day at 08:30, 10:30, 12:30, 14:30, 16:30, and 18:30 h. At those times, the exterior lights were turned off to leave the arena in the dark, and the LEDs of the shelter were turned on for 5 minutes, after which the setup was returned to the original state of illumination. In the Undisturbed condition (N = 7), none of the shelters were disturbed (see ref. 33 for more details).

### Analysis

We analyzed the RFID data to highlight the influence of disturbances on the nocturnal behavior and migration dynamics of cockroaches. For our analysis, we used parametric tests whenever the data met the normality and homogeneity of variance assumptions; otherwise, nonparametric tests were used. The R software (R Core Team, 2015), Graphpad Prism (Prism version 6.01©), and Python 3.4.1 (Python Software Foundation) platforms were used for the statistical and modeling analyses. The significance level was fixed at 0.05 for all of the tests.

To analyze the behavioral differences during the night between the Disturbed and Undisturbed conditions, we used the Kolmogorov-Smirnov test (KS test). The Total Time Outside (TTO) and the number of visits to the shelters by each individual during the active phase were used to measure the exploratory behavior. Moreover, we considered the precise moment that each individual left the shelter for the first time and the duration of this exit in further analyses.

To analyze the presence of personalities within groups, or the stability and repeatability of individual behavioral traits, we used the Kendall’s coefficient of concordance (W)^57^. This test compares the stability of rank positions for each individual within trials and provides the W coefficient, which ranges from 0 (no concordance of ranks) to 1 (complete concordance). Because a general qualitative significance threshold is not available for all situations, we compared the observed W coefficients with the “Kendall Random Distribution” (KRD) as explained in Planas-Sitjà *et al*.^42^. The KRD is the theoretical distribution of W coefficients for random rank orders of the same number of individuals and repetitions (N = 1000). We performed a Z-test to test the significance between differences in the observed W coefficient distribution and the corresponding KRD^42^,^58^. In addition, we used a linear regression model to show the relationship between variables that resulted from the same active/resting period in both conditions.

Finally, we used a probabilistic model to predict the cockroaches’ behavior throughout the week (see Fig. 2). With this model, we were able to test for the presence and influence of different personalities with regard to the emigration dynamics. The procedure and parameters are explained in The Results section. The KS test was used to analyze deviances from the theoretical distribution obtained by the model and the experimental distributions.

## Additional Information

**How to cite this article:** Planas-Sitjà, I. *et al*. Emigration dynamics of cockroaches under different disturbance regimes do not depend on individual personalities. *Sci. Rep.* **7**, 44528; doi: 10.1038/srep44528 (2017).

**Publisher's note:** Springer Nature remains neutral with regard to jurisdictional claims in published maps and institutional affiliations.

## Supplementary Material

Supplementary Information (1 and 2)

## Figures and Tables

**Table 1:** Summary of Kendall’s W values.

Group	TTO		How many times out		1st exit (duration)		1st exit (when)	
	UD	D	UD	D	UD	D	UD	D
A	0.84^***^	0.91^***^	0.79^***^	0.76^***^	0.67^***^	0.74^***^	0.44^**^	0.70^***^
B	0.79^***^	0.83^***^	0.63^***^	0.73^***^	0.73^***^	0.61^***^	0.70^***^	0.72^***^
C	0.84^***^	0.86^***^	0.67^***^	0.87^***^	0.41^*^	0.71^***^	0.53^***^	0.54^***^
D	0.84^***^	0.79^***^	0.87^***^	0.73^***^	0.79^***^	0.50^**^	0.64^***^	0.61^***^
E	0.94^***^	0.66^***^	0.90^***^	0.69^***^	0.77^***^	0.55^***^	0.50^**^	0.60^***^
F	0.77^***^	0.56^***^	0.69^***^	0.72^***^	0.65^***^	0.47^**^	0.29^**^	0.45^**^
G	0.88^***^	—	0.88^***^	—	0.79^***^	—	0.72^***^	—

UD is the Undisturbed condition and D the Disturbed condition. P-values: ***<0.0001; **<0.005; *<0.05; ns >0.05.

**Table 2:** P values for the comparison of the experimental TTO distributions amongst all transitions between the S and NS shelters in the D condition (Mann-Whitney test).

*Transitions*:	NS to NS (N = 138)	S to S (N = 209)	S to NS (N = 77)
S to S	0.22	—	—
S to NS	0.31	0.72	—
NS to S (N = 56)	0.54	0.72	0.70
