# Supplementary material for: Emigration dynamics of cockroaches under different disturbance regimes do not depend on individual personalities
Source: Sci Rep. 2017 Mar 16;7:44528. doi: 10.1038/srep44528 (PMC5353543; doi:10.1038/srep44528)
Supplement: Supplementary Information (1 and 2) [file srep44528-s1.pdf]

GROUP SELECTION DURING EMIGRATION DYNAMICS: THE ROLE OF SOCIALITY AND PERSONALITY

Planas-Sitjà *[1], I.; Laurent Salazar[1], M.O.; Sempo[1], G.; Deneubourg[1], J.L.

[1] *Unit of Social Ecology - CP 231, Université libre de Bruxelles (ULB), Campus Plaine, Boulevard du Triomphe, Building NO - level 5, B-1050 Bruxelles, Belgium*

** Author for correspondence (*isaac.planas.sitja@ulb.ac.be*).*

All the authors declare that there is no competing financial interest in relation to the work described here below.

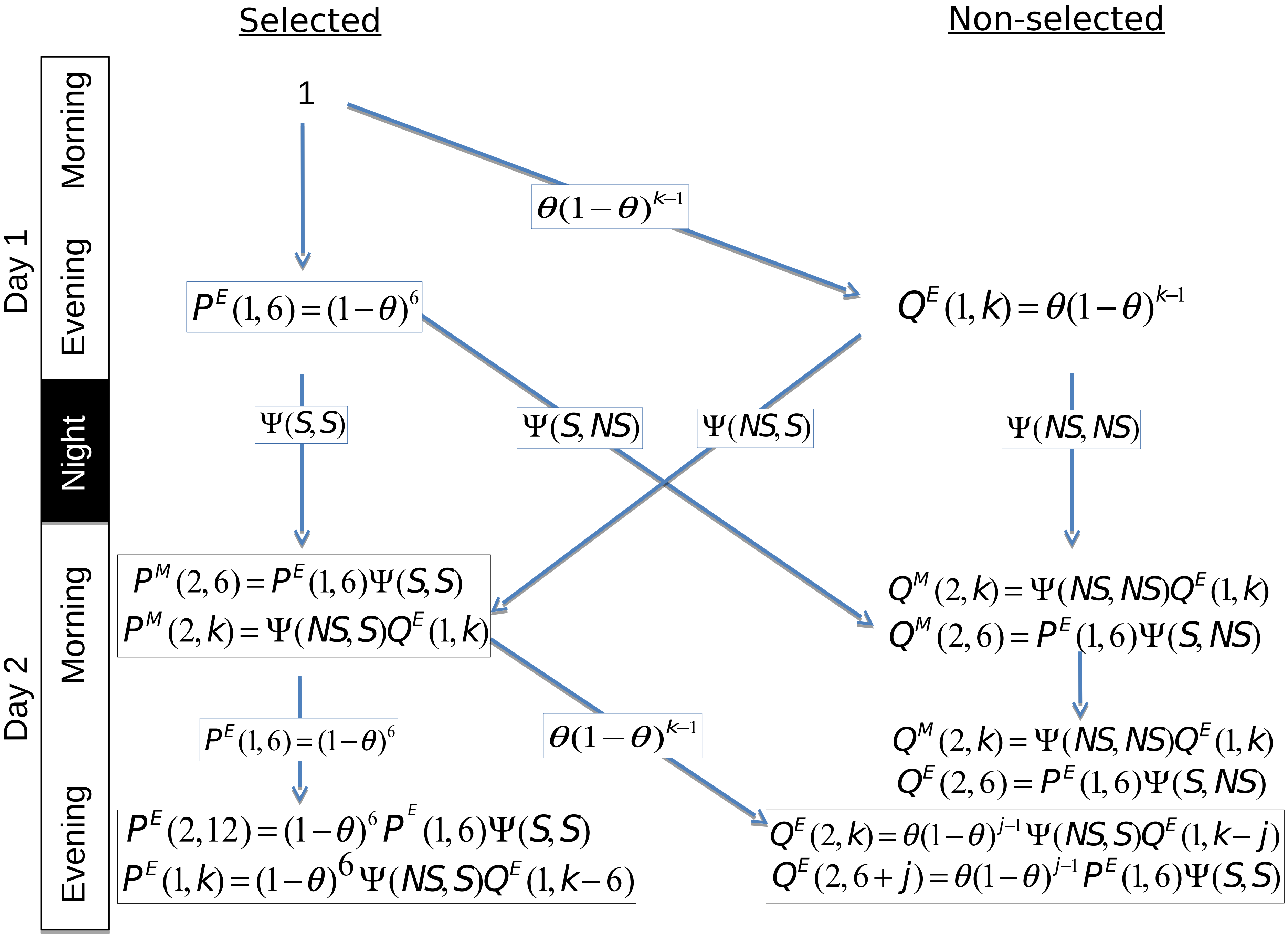
Selected
Non-selected
Day 1
Day 2
Morning
Evening
Night
Morning
Evening
1
$\theta(1-\theta)^{k-1}$
$P^E(1,6)=(1-\theta)^6$
$Q^E(1,k)=\theta(1-\theta)^{k-1}$
$\Psi(S,S)$
$\Psi(S,NS)$
$\Psi(NS,S)$
$\Psi(NS,NS)$
$P^M(2,6)=P^E(1,6)\Psi(S,S)$
$P^M(2,k)=\Psi(NS,S)Q^E(1,k)$
$Q^M(2,k)=\Psi(NS,NS)Q^E(1,k)$
$Q^M(2,6)=P^E(1,6)\Psi(S,NS)$
$P^E(1,6)=(1-\theta)^6$
$\theta(1-\theta)^{k-1}$
$Q^M(2,k)=\Psi(NS,NS)Q^E(1,k)$
$Q^E(2,6)=P^E(1,6)\Psi(S,NS)$
$P^E(2,12)=(1-\theta)^6 P^E(1,6)\Psi(S,S)$
$P^E(1,k)=(1-\theta)^6\Psi(NS,S)Q^E(1,k-6)$
$Q^E(2,k)=\theta(1-\theta)^{j-1}\Psi(NS,S)Q^E(1,k-j)$
$Q^E(2,6+j)=\theta(1-\theta)^{j-1}P^E(1,6)\Psi(S,S)$

Figure SI 1 shows a diagram describing the probability of settling in the S shelter (P^E (n,k)) or NS shelter (Q^E (n,k)) at the end of day n (evening) after experiencing k disturbances. We assume that at the beginning of the experiment (morning of the day 1), all the individuals are in the selected shelter.

First, regarding the NS shelter the cockroaches never spontaneously left the shelters during the resting phase; therefore, the probability of being at the end of the day n in the NS shelter is equal to their probability of being there at the morning.

Secondly, the probability of moving from the S shelter to the NS shelter after the kth disturbance is:

$$\theta(1-\theta)^{k-1}$$

θ is the conditional probability of moving between the S and NS shelter for each disturbance.

<u>First day evening</u>

At the end of the first day, the probability of settling in the S shelter is

$$P^E(1,6)=(1-\theta)^6$$

and *6* is the number of disturbances per day.

The probability of being in the NS shelter at the end of the first day, having been submitted to k disturbances is:

$$Q^E(1,k)=\theta(1-\theta)^{k-1}, k=1,\ldots,k$$

<u>Day 2 morning</u>

During the active phase (nighttime), the probabilities of moving to the opposite shelter are Ψ(S,NS) and Ψ(NS,S). Ψ(S, S) and Ψ(NS,NS) represent the probabilities of not moving between both shelters.

Therefore the probability of being in the S shelter the next morning, having been submitted to 6 disturbances is:

$$P^M(2,6)=\Psi(S,S)\,P^E(1,6)+\Psi(\text{NS},S)\,Q^E(1,6)$$

The first term is the probability of staying in the S shelter at the first evening and not moving during night, while the second gives the probability of leaving the S shelter only after the 6th disturbances and coming back to the S shelter during the night. In addition, the probability of having been submitted to k (=1,...,5) disturbances (day 1) and being the next morning (day 2) in S shelter is:

$$P^M(2,k)=\Psi(\text{NS},S)\,Q^E(1,k), k=1,\ldots,5$$

The probabilities to be in the NS shelter having been submitted to 1,...,6 disturbances is:

$$Q^M(2,i)=\Psi(\text{NS},\text{NS})\,Q^E(1,i), i=1,\ldots,6$$

It corresponds to individuals who move after 1,...,6 disturbances and show fidelity to the NS shelter during the night. For those who do not move during the day, but move from the S to NS shelter during the night:

$$Q^M(2,6) = \Psi(S,\mathrm{NS}) P^E(1,6)$$

<u>Day 2 evening</u>

The probability of being in the S shelter at the end of day 2 is equal to the probability of being there at the morning of day 2 and not leaving it during the day:

$$P^E(2,k) = (1-\theta)^6 P^M(1,k-6), k=7,\ldots,12$$

The probability of being in the NS shelter with *k* disturbances is the sum of the two following events:

a. The individuals which were in the NS shelter at the morning:

$$Q^E(2,k) = Q^M(2,k)$$

b. The individuals which were in the S shelter at the morning and moved to the NS shelter due to the disturbances:

$$Q^E(2,k) = \theta(1-\theta)^{j-1} P^M(1,k-j), j=1,\ldots,k$$

The same equations can be written for day 3, 4 and so on.

It is easy to get rid of the probabilities $P^M(n,k), Q^M(n,k)$, keeping only the probabilities $P^E(n,k), Q^E(n,k)$, and to write the system of equations for each evening for day n=1,..., as below:

$$P^E(\mathrm{n,k}) = \left(\Psi(\mathrm{S,S}) P^E(n-1,k-l) + \Psi(\mathrm{NS,S}) Q^E(n-1,k-l)\right)(1-\theta)^l \quad (6,a)$$

$$Q^E(\mathrm{n,k}) = \Psi(\mathrm{NS,NS}) Q^E(n-1,k) + \Psi(\mathrm{S,NS}) P^E(n-1,k) + \mathrm{A} \quad (6,b)$$

$$\mathrm{A} = \sum_{\mathrm{i=1}}^{\max(\mathrm{k,l})} \left(\Psi(\mathrm{S,S}) P^E(n-1,k-i) + \Psi(\mathrm{NS,S}) Q^E(n-1,k-i)\right)\theta(1-\theta)^{i-1} \quad (6,c)$$

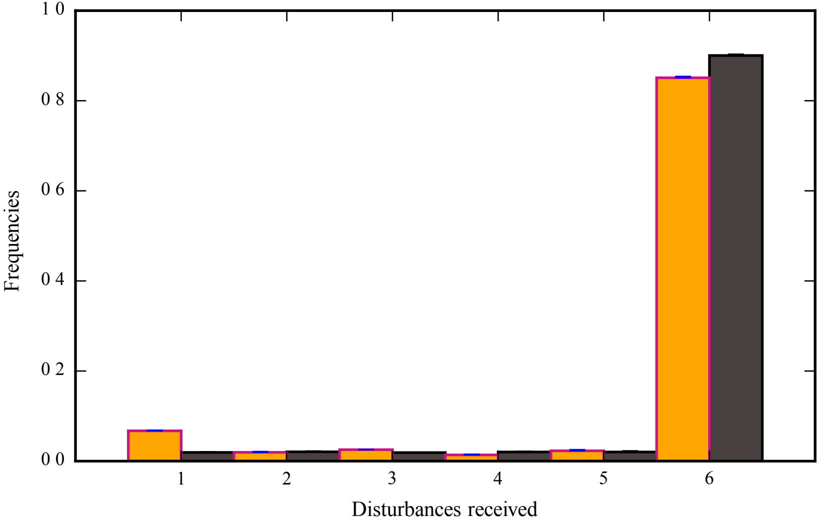

SI2 . Frequency of the mean number of disturbances experienced per day by an individual (± SD). Grey bars for simulation and yellow bars for D condition.
